# Supplementary material for: Appetite Regulation, Growth Performances and Fish Quality Are Modulated by Alternative Dietary Protein Ingredients in Gilthead Sea Bream (Sparus aurata) Culture
Source: Animals (Basel). 2021 Jun 28;11(7):1919. doi: 10.3390/ani11071919 (PMC8300235; doi:10.3390/ani11071919)
Supplement: Supplementary file 1 [file animals-11-01919-s001.zip › animals-1280587-supplementary.pdf]

Table S1. Fatty acids profile (g of FAME 100 g^{-1} total FAME) of the experimental diets. Data are expressed as mean±dev.st. obtained by 3 replicates.

	**CV**	**CF**	**H10**	**H20**	**H40**	**P20**	**P40**	**HP**	**RC10**	**MA10**
C12:0	0.09±0.02	0.09±0.01	1.42±0.20	2.70±0.28	4.11±0.40	0.10±0.02	0.10±0.02	1.40±0.20	0.10±0.04	0.13±0.02
C14:0	2.42±0.17	4.09±0.15	2.75±0.13	3.00±0.08	3.25±0.18	2.47±0.14	2.59±0.14	2.86±0.14	2.45±0.15	2.63±0.10
C16:0	14.76±0.48	17.34±0.01	14.79±0.19	14.51±0.06	14.21±0.29	15.18±0.34	15.70±0.26	15.39±0.20	14.67±0.39	15.32±0.08
C16:1n-7	2.69±0.10	4.89±0.09	2.94±0.08	3.04±0.05	3.11±0.07	3.18±0.09	3.68±0.10	3.61±0.09	3.03±0.11	3.30±0.08
C18:0	3.40±0.05	4.66±0.17	3.39±0.07	3.32±0.14	3.20±0.07	3.77±0.05	4.27±0.10	4.01±0.07	3.34±0.04	3.21±0.11
C18:1n-9	31.03±0.62	23.82±0.69	30.45±0.74	29.86±1.19	29.45±0.70	30.66±0.52	30.92±0.74	29.76±0.63	31.74±0.49	28.51±0.92
C18:1n-7	2.33±0.11	2.80±0.11	2.36±0.13	2.34±0.16	2.32±0.13	2.39±0.10	2.49±0.12	2.43±0.10	2.43±0.10	2.37±0.13
C18:2n-6	17.40±0.17	9.19±0.11	16.42±0.18	15.96±0.12	15.68±0.26	16.65±0.14	15.95±0.17	15.72±0.17	16.77±0.20	19.27±0.27
C18:3n-3	10.55±0.07	8.98±0.14	9.59±0.03	9.47±0.17	8.30±0.06	9.19±0.03	7.63±0.07	8.08±0.03	9.77±0.04	9.93±0.13
C18:4n-3	0.85±0.01	1.25±0.04	0.86±0.02	0.85±0.06	0.86±0.04	0.88±0.00	0.88±0.03	0.89±0.03	0.87±0.01	1.10±0.04
C20:1n-9	0.94±0.01	1.62±0.03	1.01±0.01	1.00±0.02	0.99±0.02	0.94±0.01	0.95±0.01	0.94±0.01	0.98±0.02	0.85±0.02
C20:5n-3	4.88±0.13	6.82±0.17	4.93±0.03	4.95±0.23	5.17±0.10	5.13±0.09	5.04±0.05	5.24±0.01	4.95±0.11	4.29±0.16
C22:6n-3	3.96±0.01	6.27±0.50	4.09±0.21	4.08±0.38	4.27±0.05	4.22±0.06	4.06±0.23	4.17±0.10	3.86±0.03	3.69±0.32
ΣSFA	21.75±0.62	27.79±0.05	23.38±0.49	24.55±0.17	25.81±0.85	22.58±0.48	23.77±0.31	24.70±0.50	21.67±0.57	22.36±0.10
ΣMUFA	38.10±0.54	35.36±1.00	38.01±0.74	37.45±1.28	37.08±0.71	38.35±0.38	39.32±0.69	38.01±0.57	39.35±0.41	36.93±0.70
Σ n-6 PUFA	17.80±0.02	10.27±0.05	16.98±0.18	16.39±0.10	16.24±0.24	17.42±0.17	17.03±0.15	16.63±0.21	17.33±0.16	19.84±0.23
Σ n-3 PUFA	21.06±0.16	24.71±0.94	20.33±0.33	20.24±0.92	19.52±0.01	20.31±0.05	18.50±0.41	19.27±0.17	20.26±0.09	19.72±0.68

CV, vegetable control; CF, fish meal control; H, *Hermetia illucens*; P, poultry by-product; RC, red swamp crayfish; MA, microalgae dried biomass.
SFA: saturated fatty acids; MUFA: monounsaturated fatty acids; PUFA: polyunsaturated fatty acids
The following FA were utilised for calculating the classes of FAs but they are not listed in the table because below 1% of total FAME: C14:1n-5, iso-C15:0, C15:0, C16:1n-9, C16:2n-4, C17:0, C16:3n-4, C17:1, C16:4n-1, C18:2n-4, C18:3n-6, C18:3n-4, C18:4n-1, C20:0, C20:1n-11, C20:1n-7, C20:2n-6, C20:3n-6, C20:4n-6, C20:3n-3, C20:4n-3, C22:0, C22:1n-11, C22:1n-9, C22:1n-7, C21:5n-3, C22:4n-6, C22:5n-6, C22:5n-3, C24:0, C24:1n-
